# Associations of serum carotenoids with visceral adiposity index and lipid accumulation product: a cross-sectional study based on NHANES 2001–2006

**DOI:** 10.1186/s12944-023-01945-6

**Published:** 2023-11-30

**Authors:** Shaohua Yan, Siyu Chen, Yumiao Liu, Hongbin Liang, Xinlu Zhang, Qiuxia Zhang, Jiancheng Xiu

**Affiliations:** Department of Cardiology, Nanfang Hospital, Southern Medical University, No. 1838, Guangzhou Avenue North, Guangzhou, 510515 China

**Keywords:** Visceral fat, Serum carotenoids, Visceral adiposity index, Lipid accumulation product, NAHNES

## Abstract

**Background:**

Visceral adiposity index (VAI) and lipid accumulation product (LAP) are comprehensive indicators to evaluate visceral fat and determine the metabolic health of individuals. Carotenoids are a group of naturally occurring antioxidants associated with several diseases. The purpose of this investigation was to explore the association between serum carotenoid concentration and VAI or LAP.

**Methods:**

The data were obtained from the National Health and Nutrition Examination Survey between 2001 and 2006. The levels of serum carotenoids were evaluated using high-performance liquid chromatography. Multivariate linear regression models were employed to investigate the relationship between levels of serum carotenoids and VAI or LAP. The potential non-linear relationship was determined using threshold effect analysis and fitted smoothing curves. Stratification analysis was performed to investigate the potential modifying factors.

**Results:**

In total, 5,084 participants were included in this population-based investigation. In the multivariate linear regressions, compared to the lowest quartiles of serum carotenoids, the highest quartiles were significantly associated with VAI, and the effect size (β) and 95% CI was − 0.98 (− 1.34, − 0.62) for α-carotene, − 1.39 (− 1.77, − 1.00) for β-carotene, − 0.79 (− 1.18, − 0.41) for β-cryptoxanthin, − 0.68 (− 0.96, − 0.39) for lutein/zeaxanthin, and − 0.88 (− 1.50, − 0.27) for trans-lycopene. Using piece-wise linear regression models, non-linear relationships were found between β­carotene and trans-lycopene and VAI with an inflection point of 2.44 (log2-transformed, ug/dL) and 3.80 (log2-transformed, ug/dL), respectively. The results indicated that α-carotene, β-cryptoxanthin, and lutein/zeaxanthin were linearly associated with VAI. An inverse association was also found between serum carotenoids and LAP after complete adjustments.

**Conclusion:**

This study revealed that several serum carotenoids were associated with VAI or LAP among the general American population. Further large prospective investigations are warranted to support this finding.

**Supplementary Information:**

The online version contains supplementary material available at 10.1186/s12944-023-01945-6.

## Introduction

Over past decades, there has been a notable increase in the worldwide prevalence of obesity, driven by alterations in dietary habits and daily lifestyles [1]. Individuals who are obese, particularly those with an excessive buildup of visceral adipose tissue, have an increased prevalence of developing hypertension, diabetes, cardiovascular diseases (CVD), and cancer [2]. The visceral adiposity index (VAI) is a valid indicator assessing the distribution and dysfunction of visceral fat in adults [3]. Unlike traditional indexes, such as waist circumference (WC) and body mass index (BMI), which primarily focus on overall weight or abdominal circumference, the VAI considers multiple factors, such as anthropometric and metabolic parameters, allowing a comprehensive evaluation of visceral fat distribution. Therefore, VAI exhibits greater sensitivity in identifying unhealthy metabolic phenotypes associated with visceral adiposity, including conditions such as insulin resistance, dyslipidemia, and cardiovascular risk factors [4–7]. The lipid accumulation product (LAP) index has garnered attention in the field of metabolic research and is used to assess and indicate the status of abdominal lipid accumulation [8]. LAP has considerable predictive capabilities compared with traditional lipid profiles for CVD, chronic kidney disease, diabetes, and other related conditions [9–11]. Studies suggest that dietary factors have a remarkable effect on obesity and lipid metabolism [12, 13].

Carotenoids are a class of lipid-soluble pigments that exhibit orange, yellow, or red colors and function as antioxidants in the human body [14]. Over 95% of the carotenoids circulating in the bloodstream comprise β-carotene, α-carotene, β-cryptoxanthin, lutein/zeaxanthin, and lycopene [15]. Several carotenoids exert a range of bioactive effects due to the antioxidant and anti-inflammatory properties [16]. Carotenoids can decrease reactive oxygen species-induced damages, prevent lipid peroxidation, and participate in cellular signaling pathways regulating apoptosis [17, 18].

Previous studies have reported inconsistent findings with respect to the effects of carotenoids on obesity and related indices. Some studies showed an inverse relationship between carotenoids and weight, adipose tissue, and anthropometric measures in obese individuals [19, 20], whereas other studies have reported no effect [21, 22]. Furthermore, limited information is available about the correlation between serum carotenoids and VAI or serum carotenoids and LAP. Elucidating the relationship between serum carotenoids and VAI or LAP could offer several novel insights into carotenoids and lipid metabolism. This study explored the correlation between levels of serum carotenoids and VAI or LAP in general United States (U.S.) adults using data from National Health and Nutrition Examination Survey (NHANES).

## Materials and methods

### Study population

**Fig. 1:**
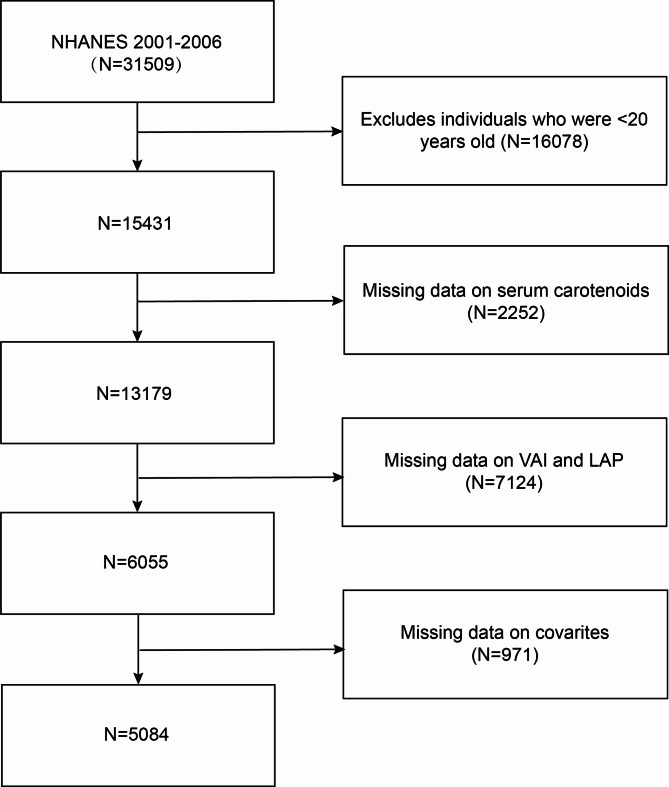
Flowchart of participant selection

NHANES is a program carried out by the National Center for Health Statistics (NCHS) aimed at gathering data on nutritional and medical conditions using a representative sample of American population. Their sampling methods use a complex and multi-stage probability approach. This study involved 15,431 individuals (age ≥ 20 years) from NHANES between 2001 and 2006. After excluding people without complete data on the five primary serum carotenoids, individuals without a reliably measured VAI or LAP were further excluded. Individuals with missing data on covariates, such as age, sex, race, alcohol intake, and smoking status, were also excluded. Lastly, this study included 5,084 participants (Fig. 1). The protocol was authorized by the NCHS Ethics Review Board and each participant gave written informed consents.

### Exposure variable and outcomes

The measurement of five serum carotenoids was performed using high-performance liquid chromatography (HPLC) [23]. Information regarding participants was available on trans-β­carotene, cis-β­carotene, α-carotene, β-cryptoxanthin, lutein/zeaxanthin, and trans-lycopene. NHANES 2001–2002 did not provide information on total lycopene. The total β­carotene level was calculated by aggregating the cis-β­carotene and trans-β­carotene concentrations. Laboratory tests were performed to calculate measurements of triglycerides (TG), high-density lipoprotein (HDL), and total cholesterol (TC) in blood samples. The VAI score was calculated using both anthropometric and biochemical data using previously established equations as reported by Amato et al [3]. LAP score was calculated using WC and TG [24].

$$Males: VAI=\left(\frac{WC}{39.68+1.88*BMI}\right)*\left(\frac{TG}{1.03}\right)*\left(\frac{1.31}{HDL}\right)$$

$$Females: VAI=\left(\frac{WC}{36.58+1.89*BMI}\right)*\left(\frac{TG}{0.81}\right)*\left(\frac{1.52}{HDL}\right)$$

$$Males: LAP=\left(WC-65\right)*TG$$

$$Females: LAP=\left(WC-58\right)*TG$$

In the equations, WC and BMI are represented in cm and kg/m^2^, respectively. The units of TG and HDL are in mmol/L.

### Covariates

Demographic data were collected via questionnaire interviews, which included age, sex, marital status, race, engagement in leisure-time physical activities, education level, and the family of poverty ratio. Weight divided by height squared was used to determine BMI. Alcohol consumption included never (< 12 drinks in lifetime), current (≥ 12 drinks and currently drinking) and former (no drink last year but ≥ 12 drinks in lifetime). Smoking status included former (≥ 100 cigarettes but not currently smoking), current (≥ 100 cigarettes and currently smoking) and never (< 100 cigarettes in lifetime). Hypertension was diagnosed according to systolic blood pressure ≥ 140 mmHg or diastolic ≥ 90 mmHg, a prior diagnosis, or a history of antihypertensive medications. Diabetes was diagnosed according to fasting glucose level (mmol/L) ≥ 7.0, glycohemoglobin (%) ≥ 6.5, the use of antidiabetic medications or insulin, or a prior diagnosis of diabetes mellitus by a physician. CVD was defined as having stroke, congestive heart failure, heart attack, angina, or coronary artery disease. All data are publicly available at www.cdc.gov/nchs/nhanes/.

### Statistical analysis

All statistical analyses followed the NHANES analytic and reporting guidelines, which involved complex survey design factors [25]. The weighted analyses were conducted with the R package “survey.” Through dividing the 2-year weights by three, new 6-year weights were obtained. Individuals were classified into four quartiles according to serum β-carotene levels, based on the abundance and high antioxidant properties of β-carotene [26, 27]. Characteristics were represented as mean ± standard error (SE) for continuous variables, and proportions were applied to describe categorical parameters. The weighted chi-square analysis and the weighted one-way analysis were performed to detect any disparities in the descriptive analyses. Multivariate linear regression models were employed to calculate size effect (β) values and 95% confidence intervals (CIs) for the association between serum carotenoid levels and VAI or LAP. No covariate was adjusted in Model (1) Age and sex were modified in Model (2) Model 3 further included race, smoking status, alcohol intake, marital status, engagement in leisure-time physical activity, BMI, the family of poverty ratio, education level, TC, hypertension, diabetes, and CVD. The smoothed curve fits were constructed to evaluate the potential non-linear relationship. We employed a threshold effect analysis model to investigate the inflection point between log2-transformed serum carotenoids and VAI or LAP. Stratification analysis was conducted to explore the potential modifying factors. The analysis was considered statistically significant if the two-sided *P*-values ≤ 0.05. We conducted statistical analyses using R Studio (Version 4.2.2) and EmpowerStats (version 4 0.1).

## Results

### Characteristics of the study population

Table 1 provides weighted baseline characteristics of participants stratified by the β-carotene quartiles. Among the 5,084 participants, the average age was 46.29 ± 0.47 years, and 2,430 (49.74%) participants were female. The average serum concentration was 4.24 ± 0.18 ug/dL for α-carotene, 8.93 ± 0.18 ug/dL for β-cryptoxanthin, 23.05 ± 0.26 ug/dL for trans-lycopene, and 15.58 ± 0.22 ug/dL for lutein/zeaxanthin.

**Table 1:** Baseline characteristics of study participants stratified by serum β-carotene quartiles

variable	Serum β-carotene (ug/dl)					*P* value
	total	Q1	Q2	Q3	Q4	
Age (year)	46.29 ± 0.47	41.82 ± 0.54	44.11 ± 0.57	47.68 ± 0.74	52.49 ± 0.69	< 0.001
Sex, n (%)						< 0.001
Female	2430 (49.74)	476 (37.74)	570 (48.47)	635 (52.42)	749 (62.38)	
Male	2654 (50.26)	796 (62.26)	703 (51.53)	637 (47.58)	518 (37.62)	
Race, n (%)						0.071
Non-Hispanic White	2742 (74.17)	675 (72.97)	689 (75.17)	653 (72.06)	725 (76.55)	
Non-Hispanic Black	969 (10.04)	295 (11.63)	241 (10.00)	226 (10.16)	207 (8.13)	
Mexican American	1035 (7.35)	213 (6.28)	262 (7.28)	303 (8.99)	257 (7.03)	
Others	338 (8.44)	89 (9.12)	81 (7.54)	90 (8.79)	78 (8.29)	
Education level, n (%)						< 0.001
Less than high school	1402 (17.19)	369 (20.54)	366 (18.62)	350 (14.88)	317 (14.05)	
High school	1251 (26.56)	374 (32.54)	327 (27.27)	307 (26.22)	243 (19.15)	
College or above	2431 (56.25)	529 (46.92)	580 (54.11)	615 (58.89)	707 (66.80)	
Marital, n (%)						< 0.001
Married	2860 (59.00)	604 (51.55)	711 (57.45)	759 (61.29)	786 (67.08)	
Separated	1463 (25.48)	410 (29.19)	345 (25.28)	344 (24.05)	364 (22.82)	
Unmarried	761 (15.52)	258 (19.27)	217 (17.27)	169 (14.66)	117 (10.10)	
Smoking status, n (%)						< 0.001
Never	2493 (48.38)	499 (38.37)	584 (45.80)	690 (54.38)	720 (56.81)	
Former	1426 (26.13)	269 (19.62)	358 (26.03)	366 (26.83)	433 (33.09)	
Now	1165 (25.49)	504 (42.01)	331 (28.17)	216 (18.79)	114 (10.10)	
Alcohol user, n (%)						< 0.001
Never	683 (11.53)	115 (7.43)	170 (11.85)	179 (12.88)	219 (14.57)	
Former	1089 (17.45)	244 (16.88)	271 (17.76)	296 (17.87)	278 (17.35)	
Now	3312 (71.02)	913 (75.69)	832 (70.40)	797 (69.25)	770 (68.08)	
Family of poverty ratio, n (%)						< 0.001
< 1.3	1286 (17.85)	412 (23.55)	331 (18.12)	314 (16.55)	229 (12.25)	
1.3–3.5	2022 (37.34)	509 (40.52)	534 (40.16)	488 (35.83)	491 (32.02)	
>3.5	1776 (44.81)	351 (35.93)	408 (41.72)	470 (47.62)	547 (55.73)	
BMI (kg/m^2^)	28.35 ± 0.12	30.09 ± 0.21	28.65 ± 0.24	28.07 ± 0.20	26.29 ± 0.18	< 0.001
Total cholesterol (mmol/L)	5.16 ± 0.02	4.91 ± 0.03	5.09 ± 0.04	5.29 ± 0.04	5.39 ± 0.04	< 0.001
Leisure-time physical activity						< 0.001
Yes	1356 (29.83)	281 (22.79)	336 (29.71)	329 (29.84)	410 (38.14)	
No	3728 (70.17)	991 (77.21)	937 (70.29)	943 (70.16)	857 (61.86)	
CVD, n (%)						0.460
Yes	576 (8.31)	132 (7.81)	153 (9.20)	131 (7.72)	160 (8.49)	
No	4508 (91.69)	1140 (92.19)	1120 (90.80)	1141 (92.28)	1107 (91.51)	
Hypertension, n (%)						0.040
Yes	2178 (36.59)	560 (39.92)	523 (36.35)	542 (35.34)	553 (34.24)	
No	2906 (63.41)	712 (60.08)	750 (63.65)	730 (64.66)	714 (65.76)	
Diabetes, n (%)						0.002
Yes	650 (8.95)	207 (11.89)	159 (8.29)	147 (7.70)	137 (7.51)	
No	4434 (91.05)	1065 (88.11)	1114 (91.71)	1125 (92.30)	1130 (92.49)	
α-carotene (ug/dL)	4.24 ± 0.18	1.28 ± 0.04	2.48 ± 0.05	4.22 ± 0.13	9.66 ± 0.45	< 0.001
β-cryptoxanthin (ug/dL)	8.93 ± 0.18	4.85 ± 0.10	7.22 ± 0.17	10.18 ± 0.24	14.33 ± 0.32	< 0.001
Lutein/zeaxanthin (ug/dL)	15.58 ± 0.22	10.82 ± 0.17	13.84 ± 0.24	16.78 ± 0.25	21.82 ± 0.42	< 0.001
Trans-lycopene (ug/dL)	23.05 ± 0.26	19.10 ± 0.34	23.43 ± 0.40	24.52 ± 0.41	25.74 ± 0.42	< 0.001

All values are presented as number and proportion (%), or mean and SE.

Compared with the quartile 1 group, participants with the highest serum β-carotene concentration were patients who were older, female, better educated, married, never smokers, inclined to participate in leisure-time physical activity, had lower BMI, higher family income–poverty ratio level, and less likely to be diagnosed with diabetes and hypertension.

### Association between serum carotenoid concentration and VAI and LAP

Three multiple regression models were conducted to determine the correlation of various carotenoids in the serum with VAI. In the crude model, the highest quartiles of five carotenoids were significantly associated with VAI compared with their respective lowest quartiles. after adjusting all covariates, the inverse association was robust between α-carotene (− 0.98 [95% CI, − 1.34 to − 0.62]), β-carotene (− 1.39, [95% CI, − 1.77 to − 1.00]), β-cryptoxanthin (− 0.79, [95% CI, − 1.18 to − 0.41]), lutein/zeaxanthin (− 0.68 (95% CI, − 0.96 to − 0.39]), trans-lycopene (− 0.88 [95% CI, − 1.50 to − 0.27]) with VAI (Table 2).

**Table 2:** The associations of the quartile of serum carotenoids, relative to Quartile 1 with VAI

Categories	Range	Model1	Model2	Model3
		β (95%CI)	β (95%CI)	β (95%CI)
**α-carotene (ug/dL)**				
Continuous		−0.29 (− 0.36, − 0.21)	−0.31 (− 0.39, − 0.23)	−0.25 (− 0.33, − 0.16)
Q1	< 1.40	ref	ref	ref
Q2	1.40–2.70	−0.47 (− 0.82, − 0.12)	−0.52 (− 0.88, − 0.16)	−0.49 (− 0.83, − 0.14)
Q3	2.70–5.00	−0.64 (− 0.95, − 0.33)	−0.73 (− 1.05, − 0.40)	−0.70 (− 1.04, − 0.36)
Q4	≥ 5.00	−0.93 (− 1.24, − 0.63)	−1.05 (− 1.38, − 0.72)	−0.98 (− 1.34, − 0.62)
*P* for trend		< 0.001	< 0.001	< 0.001
**β-carotene (ug/dL)**				
Continuous		−0.35 (− 0.47, − 0.24)	−0.41 (− 0.54, − 0.27)	−0.32 (− 0.44, − 0.20)
Q1	< 7.50	ref	ref	ref
Q2	7.50–12.89	−0.59 (− 0.93, − 0.26)	−0.65 (− 0.99, − 0.30)	−0.62 (− 0.97, − 0.26)
Q3	12.89–23.30	−0.73 (− 1.01, − 0.46)	−0.86 (− 1.15, − 0.57)	−0.85 (− 1.17, − 0.54)
Q4	> 23.30	−1.28 (− 1.60, − 0.96)	−1.52 (− 1.87, − 1.17)	−1.39 (− 1.77, − 1.00)
*P* for trend		< 0.001	< 0.001	< 0.001
**β-cryptoxanthin (ug/dL)**				
Continuous		−0.26 (− 0.38, − 0.14)	−0.27 (− 0.39, − 0.14)	−0.28 (− 0.42, − 0.14)
Q1	< 4.80	ref	ref	ref
Q2	4.80–7.70	−0.42 (− 0.77, − 0.08)	−0.41 (− 0.75, − 0.07)	−0.42 (− 0.76, − 0.09)
Q3	7.70–12.70	−0.60 (− 0.89, − 0.32)	−0.60 (− 0.88, − 0.32)	−0.57 (− 0.90, − 0.24)
Q4	> 12.70	−0.76 (− 1.08, − 0.43)	−0.78 (− 1.10, − 0.45)	−0.79 (− 1.18, − 0.41)
*P* for trend		< 0.001	< 0.001	< 0.001
**Lutein/zeaxanthin (ug/dL)**				
Continuous		−0.18 (− 0.28, − 0.08)	−0.23 (− 0.32, − 0.13)	−0.28 (− 0.39, − 0.16)
Q1	< 10.46	ref	ref	ref
Q2	10.46–14.50	−0.09 (− 0.41, 0.22)	−0.12 (− 0.43, 0.19)	−0.19 (− 0.46, 0.09)
Q3	14.50–20.10	−0.42 (− 0.62, − 0.21)	−0.47 (− 0.68, − 0.27)	−0.54 (− 0.79, − 0.29)
Q4	≥ 20.10	−0.40 (− 0.64, − 0.16)	−0.51 (− 0.75, − 0.27)	−0.68 (− 0.96, − 0.39)
*P* for trend		< 0.001	< 0.001	< 0.001
**Trans-lycopene (ug/dL)**				
Continuous		−0.12 (− 0.24, 0.00)	−0.08 (− 0.21, 0.04)	−0.33 (− 0.53, − 0.12)
Q1	< 14.00	ref	ref	ref
Q2	14.00–20.11	−0.48 (− 0.85, − 0.09)	−0.39 (− 0.78, 0.00)	−0.38 (− 0.81, 0.04)
Q3	20.11–28.23	−0.39 (− 0.79, 0.00)	−0.28 (− 0.70, 0.14)	−0.46 (− 0.96, 0.04)
Q4	≥ 28.23	−0.48 (− 0.87, − 0.10)	−0.38 (− 0.78, 0.02)	−0.88 (− 1.50, − 0.27)
*P* for trend		0.023	0.104	0.005

Model 1: no cofounder; Model 2: adjusted for age, sex; Model 3: further adjusted for race, education level, marital status, alcohol intake, smoking status, leisure-time physical activity, BMI, the family of poverty ratio, TC, hypertension, diabetes, and CVD.

Five carotenoids were negatively correlated with LAP in the crude model (Table 3). When serum carotenoids were calculated as continuous variables, multivariate regression analysis revealed an inverse correlation between β-carotene, α-carotene, β-cryptoxanthin, trans-lycopene, and LAP. When they were divided into quartiles, the β values and 95% CIs of participants in fourth quartiles were (− 19.40 [− 25.47, − 13.32]) for α-carotene, (− 29.21 [− 35.21, − 23.22]) for β-carotene, (− 13.82 [− 19.59, − 8.05]) for β-cryptoxanthin, (− 10.11 [− 15.73, − 4.50]) for lutein/zeaxanthin, (− 15.65 [− 24.09, − 7.21]) for trans-lycopene after complete adjustment compared with the lowest quartile (Table 3).

**Table 3:** The associations of the quartile of serum carotenoids, relative to Quartile 1 with LAP

Categories		Model1	Model2	Model3
		β (95%CI)	β (95%CI)	β (95%CI)
**α-carotene (ug/dL)**				
Continuous		−8.52 (− 10.44, − 6.59)	−8.97 (− 11.13, − 6.81)	−4.47 (− 6.10, − 2.84)
Q1	< 1.40	Ref	Ref	Ref
Q2	1.40–2.70	−12.25 (− 19.59, − 4.90)	−13.67 (− 21.18, − 6.15)	−10.46 (− 16.56, − 4.35)
Q3	2.70–5.00	−17.54 (− 24.88, − 10.19)	−20.11 (− 27.56, − 12.66)	−14.49 (− 21.13, − 7.85)
Q4	≥ 5.00	−27.71 (− 34.36, − 21.06)	−30.63 (− 37.71, − 23.54)	−19.40 (− 25.47, − 13.32)
*P* for trend		< 0.001	< 0.001	< 0.001
**β-carotene (ug/dL)**				
Continuous		−10.46 (− 13.49, − 7.43)	−11.98 (− 15.57, − 8.39)	−6.18 (− 8.13, − 4.22)
Q1	< 7.50	Ref	Ref	Ref
Q2	7.50–12.89	−18.45 (− 25.51, − 11.38)	−19.52 (− 26.63, − 12.41)	−14.50 (− 21.00, − 7.99)
Q3	12.89–23.30	−21.99 (− 28.51, − 15.46)	−25.67 (− 32.11, − 19.23)	−18.84 (− 24.33, − 13.35)
Q4	> 23.30	−37.83 (− 43.99, − 31.67)	−44.69 (− 51.25, − 38.13)	−29.21 (− 35.21, − 23.22)
*P* for trend		< 0.001	< 0.001	< 0.001
**β-cryptoxanthin (ug/dL)**				
Continuous		−8.56 (− 11.19, − 5.92)	−8.71 (− 11.42, − 5.99)	−4.87 (− 7.05, − 2.69)
Q1	< 4.80	Ref	Ref	Ref
Q2	4.80–7.70	−12.11 (− 20.06, − 4.17)	−11.32 (− 19.19, − 3.46)	−8.55 (− 14.71, − 2.40)
Q3	7.70–12.70	−17.88 (− 24.71, − 11.04)	−17.58 (− 24.07, − 11.09)	−10.72 (− 16.28, − 5.15)
Q4	> 12.70	−24.4 (− 30.58, − 18.23)	−24.83 (− 30.91, − 18.75)	−13.82 (− 19.59, − 8.05)
*P* for trend		< 0.001	< 0.001	< 0.001
**Lutein/zeaxanthin (ug/dL)**				
Continuous		−4.94 (− 7.31, − 2.57)	−6.88 (− 9.26, − 4.51)	−4.29 (− 6.35, − 2.22)
Q1	< 10.46	Ref	Ref	Ref
Q2	10.46–14.50	−2.2 (− 9.00, 4.61)	−3.63 (− 10.25, 2.98)	−2.76 (− 7.82, 2.31)
Q3	14.50–20.10	−10.17 (− 15.38, − 4.97)	−12.78 (− 17.71, − 7.85)	−9.00 (− 13.64, − 4.37)
Q4	≥ 20.10	−10.39 (− 16.33, − 4.46)	−15.3 (− 21.00, − 9.60)	−10.11 (− 15.73, − 4.50)
*P* for trend		< 0.001	< 0.001	< 0.001
**Trans-lycopene (ug/dL)**				
Continuous		−1.55 (− 4.01, 0.91)	−0.16 (− 2.74, 2.42)	−5.88 (− 8.62, − 3.13)
Q1	< 14.00	Ref	Ref	Ref
Q2	14.00–20.11	−9.74 (− 16.13, − 3.35)	−5.51 (− 11.88, 0.85)	−5.84 (− 12.49, 0.81)
Q3	20.11–28.23	−8.04 (− 15.57, − 0.50)	−2.49 (− 10.37, 5.38)	−6.87 (− 14.42, 0.67)
Q4	≥ 28.23	−8.55 (− 15.67, − 1.43)	−3.79 (− 11.20, 3.63)	−15.65 (− 24.09, − 7.21)
*P* for trend		0.051	0.534	< 0.001

Model 1: no cofounder; Model 2: adjusted for age, sex; Model 3: further adjusted for race, education level, marital status, alcohol intake, smoking status, leisure-time physical activity, BMI, the family of poverty ratio, TC, hypertension, diabetes, and CVD.

**Fig. 2:**
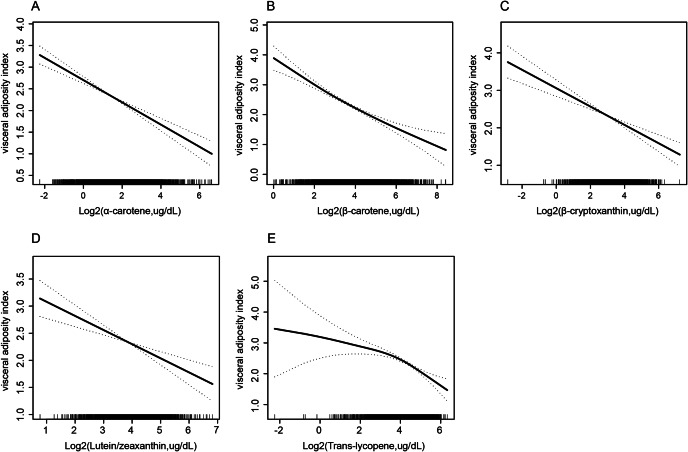
Association of specific serum Carotenoids with visceral adiposity index (α-carotene **(A)**, β-carotene **(B)**, β-cryptoxanthin **(C)**, Lutein/zeaxanthin **(D)**, Trans-lycopene **(E)**). Adjusted for age, sex, race, education level, marital status, alcohol intake, smoking status, leisure-time physical activity, BMI, the family of poverty ratio, TC, hypertension, diabetes, and CVD

Piece-wise linear regression models revealed non-linear relationships between β­carotene and trans-lycopene and VAI with an inflection point of 2.44 (log2-transformed, ug/dL) and 3.80 (log2-transformed, ug/dL), respectively. The results indicated that α-carotene, β-cryptoxanthin, and lutein/zeaxanthin were linearly related to VAI. (Table 4; Fig. 2A–E)

**Table 4:** Threshold effect analysis of serum carotenoids on VAI using piece-wise linear regression

VAI		Adjusted β (95% CI) *P* value
**α-carotene**		
One line slop		−0.26 (− 0.31, − 0.20) < 0.0001
Log likelihood ratio test		0.071
**β-carotene**		
Inflection point (K)		2.44
<K slope		−0.63 (− 0.89, − 0.37) < 0.0001
>K slope		−0.33 (− 0.40, − 0.25) < 0.0001
Log likelihood ratio test		0.038
**β-cryptoxanthin**		
One line slop		−0.24 (− 0.32, − 0.17) < 0.0001
Log likelihood ratio test		0.447
**Lutein/zeaxanthin**		
One line slop		−0.26 (− 0.37, − 0.15) < 0.0001
Log likelihood ratio test		0.354
**Trans-lycopene**		
Inflection point (K)		3.80
<K slope		−0.11 (− 0.27, 0.06) 0.1966
>K slope		−0.47 (− 0.62, − 0.33) < 0.0001
Log likelihood ratio test		0.005

Adjusted for age, sex, race, education level, marital status, alcohol intake, smoking status, leisure-time physical activity, BMI, the family of poverty ratio, TC, hypertension, diabetes, and CVD.

**Fig. 3:**
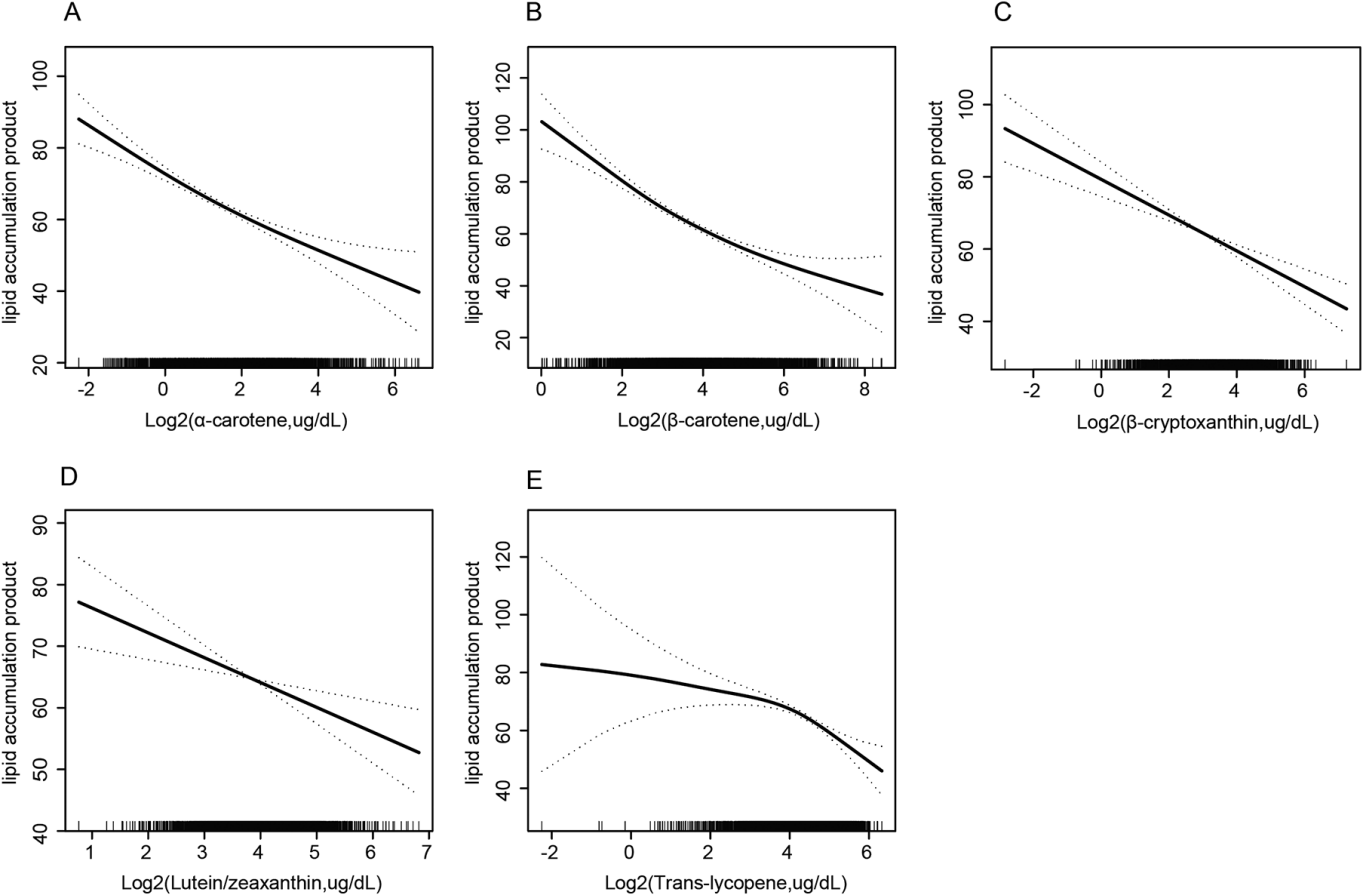
Association of specific serum Carotenoids with lipid accumulation product (α-carotene **(A)**, β-carotene **(B)**, β-cryptoxanthin **(C)**, Lutein/zeaxanthin **(D)**, Trans-lycopene **(E)**). Adjusted for age, sex, race, education level, marital status, alcohol intake, smoking status, leisure-time physical activity, BMI, the family of poverty ratio, TC, hypertension, diabetes, and CVD

Similarly, Table 5 showed that β-cryptoxanthin and lutein/zeaxanthin were linearly related to LAP, whereas α-carotene, β­carotene and trans-lycopene were non-linearly related to LAP with an inflection point of − 0.51 (log2-transformed, ug/dL), 2.93 (log2-transformed, ug/dL), and 4.29 (log2-transformed, ug/dL), respectively (Table 5; Fig. 3A–E).

**Table 5:** Threshold effect analysis of serum carotenoids on LAP using piece-wise linear regression

LAP		Adjusted β (95% CI) *P* value
**α-carotene**		
Inflection point (K)		−0.51
<K slope		−15.17 (− 24.33, − 6.02) 0.0012
>K slope		−4.94 (− 6.30, − 3.58) < 0.0001
Log likelihood ratio test		0.038
**β-carotene**		
Inflection point (K)		2.93
<K slope		−13.56 (− 17.46, − 9.67) < 0.0001
>K slope		−6.52 (− 8.31, − 4.74) < 0.0001
Log likelihood ratio test		0.004
**β-cryptoxanthin**		
One line slop		−4.95 (− 6.57, − 3.34) < 0.0001
Log likelihood ratio test		0.253
**Lutein/zeaxanthin**		
One line slop		−4.03 (− 6.38, − 1.69) 0.0008
Log likelihood ratio test		0.367
**Trans-lycopene**		
Inflection point (K)		4.29
<K slope		−2.66 (− 5.45, 0.13) 0.0613
>K slope		−12.00 (− 16.38, − 7.63) < 0.0001
Log likelihood ratio test		0.002

Adjusted for age, sex, race, education level, marital status, alcohol intake, smoking status, leisure-time physical activity, BMI, the family of poverty ratio, TC, hypertension, diabetes, and CVD.

### Sensitivity analyses

Stratified analyses were performed to investigate the relation of specific serum carotenoids (per SD increment) with VAI or LAP. No significant interaction was found when data were stratified by sex, race, BMI, alcohol intake, smoking status, hypertension, and CVD (Supplementary Tables 1–5). Consistent outcomes were obtained when current smokers were excluded (Supplemental Table 6).

## Discussion

This population-based study revealed that higher levels of serum carotenoids, including α-carotene, β-carotene, β-cryptoxanthin, lutein/zeaxanthin, and trans-lycopene, are correlated with lower VAI or LAP. Non-linear relationships were found among certain serum carotenoids and VAI or LAP. No significant interactions were found in subgroup analyses.

The dysregulation of lipid homeostasis is considered a common characteristic in several diseases, particularly metabolic disorders. Changes in lipid profiles often occur before the onset of diseases [28, 29]. Obesity-related physiological abnormalities are predominantly affected by the distribution of body fat, rather than solely attributed to the presence of overweight or obesity [30–32]. Notably, previous studies have reported a strong association between visceral fat, rather than subcutaneous fat, and metabolic risk factors [31].

In the sensitive detection of visceral fat, techniques such as computed tomography and magnetic resonance imaging are widely used. However, these methods have limitations such as high costs, time-consuming procedures, and potential radiation hazards. As a result, these techniques are not feasible for large-scale population screenings. Traditional indicators, including BMI and WC, can reflect the degree of overweight but they are limited in the capacity to assess fat distribution. Conversely, VAI and LAP have been recognized as novel markers for evaluating visceral fat in a simple and noninvasive manner. Unlike traditional lipid profiles, VAI and LAP can assess many metabolic disorder syndromes and provide a comprehensive assessment of the metabolic health of individuals [33–36].

The inverse correlation between serum carotenoids and VAI or LAP could be due to the antioxidant properties of carotenoids [37]. Oxidative stress increases the accumulation of white adipose tissue (WAT), stimulation of preadipocyte proliferation and differentiation, and enlargement of mature adipocytes [38]. Carotenoids play an important role in oxidative metabolism by inhibiting lipid peroxidation and participating in cell interactions involved in apoptosis [17, 18, 37]. Further, carotenoids are recognized as precursors to retinoids, which are considered to block the formation of adipocytes and decrease fat accumulation [39]. Retinoids inhibit the activation of peroxisome proliferator-activated receptor γ, a critical transcription factor required for fat accumulation in adipocytes [40]. Additionally, retinoic acid stimulates the upregulation of uncoupling protein-1 gene expression, which is crucial for facilitating the uncoupling of mitochondrial respiration [41, 42]. This plays an important role in decreasing fat accumulation within WAT [43]. Carotenoids can regulate insulin resistance and promote insulin secretion to decrease abdominal fat accumulation through the regulation of hormone-sensitive lipase [22, 44]. In this study, the adverse connection between five serum carotenoids and metabolic indicators, such as VAI and LAP, may be due to the aforementioned underlying mechanism. To conclude, carotenoids play a vital role in various stages of the lipid metabolic process.

Previous study has showed the correlation between carotenoids and obesity, obesity indices, and obesity-related diseases, across different epidemiological methodologies and target populations. Several epidemiological and observational studies have reported that both young and old people with obesity have lower plasma carotenoid concentrations [45–47]. Furthermore, levels of adipose carotenoids obtained from the buttock, abdomen, and inner thigh showed an inverse correlation with body fat mass [48]. β-carotene content in adipocytes collected from obese individuals was approximately half of that collected from individuals with normal weight [49]. In the Coronary Artery Risk Development in Young Adults study over seven years, the connection between the change in serum carotenoids (excluding lycopene) and the change in BMI was inversely related among non-smokers; however, this correlation was not observed among smokers [50]. A previous study conducted in the U.S. females also revealed a negative association between dietary lutein/zeaxanthin intake and metabolic syndrome, which is strongly associated with obesity and dyslipidemia [51]. Another intervention pilot study performed in obese middle-aged Japanese men with a BMI of ≥ 25 kg/m^2^ indicated that short-term consumption of lycopene and lutein decreased the intra-abdominal visceral fat, which is consistent with the adverse association between serum carotenoids and visceral fat indicators of this research [52]. However, the small sample size and lack of survey of other dietary intake restricted their outcomes.

In some interventional trials, the results showed carotenoids had no effect [20–22]. In healthy Japanese males, limited associations were found between obesity indicators, such as WC and waist-to-hip ratio and serum concentration of carotenoids including α­carotene and β­carotene [20]. This can be because approximately 70% of male participants in the study were heavy smokers, consuming over 20 cigarettes per day. The absence of a statistically significant correlation between the variables was most likely caused by the considerable effect of smoking on the blood concentration of carotenoids and obesity indices. Consistent with the the aforementioned studies, this study showed an inverse correlation between specific serum concentrations of carotenoids and VAI and LAP in a large-scale and normal U.S. population based on another metabolic trait and obesity phenotype different from unconventional indices.

### Strengths and limitations

The *p*resent study has multiple strengths. The results were derived from a substantial, nationally representative sample, allowing for the weighted outcomes that reflect the U.S. population at the national level. Furthermore, a broad range of potential confounding factors were considered in this study and subgroup analyses were conducted to ensure consistent results. This study has limitations that should be addressed. We were incapable of establishing a causal relationship based on a cross-sectional study design, not longitudinal. Additionally, although several covariates were considered, there may still be residual confounders affecting the outcomes. More investigations are required to highlight the effect of smoking status on the concentration of serum carotenoids. Moreover, the relationship between total serum carotenoids and obesity indicators could not be established due to the incomplete total lycopene. Lastly, considering the complex metabolism of serum carotenoids in vivo, additional research should be taken into consideration.

## Conclusion

To summarize, this study conducted was on the sample obtained from the nationally representative U.S. population. An inverse relationship was found between VAI or LAP and the serum concentrations of carotenoids after complete adjustments. The findings have potential public health implications and support the metabolic benefits of serum carotenoids on obesity and lipid metabolism in adults. However, to validate the causal relationship and elucidate the underlying mechanism, further investigations are required.

### Electronic supplementary material

Below is the link to the electronic supplementary material.

Supplementary Material 1

Supplementary Material 2

Supplementary Material 3

## Data Availability

The survey data are publicly available on the internet for data users and researchers throughout the world (www.cdc.gov/nchs/nhanes/).

## Abbreviations

VAI: Visceral adiposity index
LAP: Lipid accumulation product
NHANES: National Health and Nutrition Examination Survey
WC: Waist circumference
BMI: Body mass index
CVD: Cardiovascular diseases
NCHS: National Center for Health Statistics
HPLC: High-performance liquid chromatography
TG: Triglycerides
HDL: High-density lipoprotein
TC: Total cholesterol
WAT: White adipose tissue
