# Supplementary material for: Associations of serum carotenoids with visceral adiposity index and lipid accumulation product: a cross-sectional study based on NHANES 2001–2006
Source: Lipids Health Dis. 2023 Nov 30;22:209. doi: 10.1186/s12944-023-01945-6 (PMC10691056; doi:10.1186/s12944-023-01945-6)
Supplement: Supplementary file 1 — Supplementary Material 1 [file 12944_2023_1945_MOESM1_ESM.pdf]

Bullet Edits

EDITORIAL CERTIFICATE

This document certifies that the paper listed below was edited and proofread for proper English language, grammar, punctuation, spelling, and overall style by one or more than one highly qualified native speakers at Bullet Edits. All of the suggested amendments were tracked with the Microsoft Word "Track Changes" feature. Therefore, the author had the option to reject or accept each change individually.

Manuscript Title:

Associations of serum carotenoids with visceral adiposity index and lipid accumulation product: A cross-sectional study based on NHANES 2001–2006

Anti-counterfeiting Code:

f90b3e365e9dc55abc3806a6cb299ba8

Date Issued:

2023-10-11 21:02:37

Bullet Edits is a registered company headquartered in the UK with a global presence. We offer a range of editing, proofreading services to authors. Our Ph.D. editors are all native English speakers from the USA and UK. Authors who work with Bullet Edits are guaranteed excellent language quality and timely delivery.

Address: BULLET EDITS LIMITED, 85 Great Portland Street, London,UK
Tel: UK. (+44)20457 70286 / US.(+1)312-313-9179
Web: www.bulletedits.cn/Email: info@bulletedits.cn
VAT: GB 378 9316 43

BULLET EDITS LIMITED ★ COMPANY NO. 13345261 SEAL ★ ENGLAND AND WALES
