# Supplementary material for: Associations of serum carotenoids with visceral adiposity index and lipid accumulation product: a cross-sectional study based on NHANES 2001–2006
Source: Lipids Health Dis. 2023 Nov 30;22:209. doi: 10.1186/s12944-023-01945-6 (PMC10691056; doi:10.1186/s12944-023-01945-6)
Supplement: Supplementary file 2 — Supplementary Material 2 [file 12944_2023_1945_MOESM2_ESM.pdf]

Supplementary Table 1. The relation of serum α-carotene concentration with VAI or LAP in various subgroups.

α-carotene	VAI		LAP	
	β (95% CI)	*P* for interaction	β (95% CI)	*P* for interaction
Age		0.066		0.034
<60	−0.23 (−0.34, −0.12)		−4.07 (−5.95, −2.18)	
≥60	−0.37 (−0.51, −0.22)		−6.88 (−10.15, −3.60)	
Sex		0.507		0.407
Male	−0.22 (−0.32, −0.13)		−4.70 (−7.15, −2.25)	
Female	−0.26 (−0.39, −0.12)		−4.29 (−6.62, −1.97)	
BMI		0.893		0.610
<30	−0.26 (−0.33, −0.18)		−5.68 (−7.11, −4.25)	
≥30	−0.25 (−0.59, 0.09)		−6.95 (−16.28, 2.37)	
Race		0.052		0.165
white	−0.28 (−0.39, −0.16)		−4.59 (−6.69, −2.48)	
non-white	−0.14 (−0.23, −0.05)		−3.47 (−5.24, −1.70)	
Current drinker		0.810		0.985
Now	−0.24 (−0.34, −0.14)		−4.50 (−6.36, −2.65)	
No	−0.26 (−0.41, −0.11)		−4.19 (−7.32, −1.05)	
Current smoker		0.225		0.526
Yes	−0.82 (−1.60, −0.05)		−11.89 (−27.33, 3.55)	
No	−0.20 (−0.27, −0.14)		−4.07 (−5.50, −2.64)	
Hypertension		0.397		0.183
Yes	−0.30 (−0.45, −0.16)		−6.18 (−9.88, −2.49)	
No	−0.22 (−0.33, −0.11)		−3.84 (−5.69, −1.99)	
CVD		0.638		0.326
Yes	−0.27 (−0.53, −0.01)		−6.07 (−12.40, 0.27)	
No	−0.25 (−0.34, −0.16)		−4.47 (−6.13, −2.81)	

Adjusted for age, sex, race, education level, marital status, alcohol intake, smoking status, leisure-time physical activity, BMI, the family of poverty ratio, TC, hypertension, diabetes, and CVD, if not stratified.

Supplementary Table 2. The relation of serum β-carotene concentration with VAI or LAP in various subgroups.

β-carotene	VAI		LAP	
	β (95% CI)	*P* for interaction	β (95% CI)	*P* for interaction
Age		0.882		0.852
<60	−0.33 (−0.50, −0.16)		−6.20 (−9.07, −3.32)	
≥60	−0.31 (−0.41, −0.20)		−5.92 (−8.16, −3.68)	
Sex		0.965		0.179
Male	−0.31 (−0.48, −0.14)		−7.37 (−11.37, −3.36)	
Female	−0.32 (−0.46, −0.17)		−5.58 (−7.83, −3.33)	
BMI		0.089		0.005
<30	−0.30 (−0.41, −0.18)		−6.76 (−9.27, −4.24)	
≥30	−0.60 (−0.94, −0.26)		−17.88 (−26.04, −9.71)	
Race		0.447		0.457
white	−0.30 (−0.43, −0.17)		−5.61 (−7.71, −3.52)	
non-white	−0.40 (−0.55, −0.25)		−8.64 (−11.48, −5.81)	
Current drinker		0.298		0.557
Now	−0.30 (−0.44, −0.16)		−6.01 (−8.47, −3.55)	
No	−0.40 (−0.54, −0.25)		−6.68 (−9.47, −3.89)	
Current smoker		0.014		0.008
Yes	−1.23 (−2.03, −0.43)		−23.07 (−35.40, −10.74)	
No	−0.26 (−0.34, −0.17)		−5.17 (−6.84, −3.50)	
Hypertension		0.454		0.091
Yes	−0.37 (−0.52, −0.22)		−7.95 (−11.05, −4.86)	
No	−0.29 (−0.44, −0.14)		−5.36 (−7.83, −2.88)	
CVD		0.934		0.793
Yes	−0.26 (−0.55, 0.03)		−5.57 (−11.70, 0.57)	
No	−0.33 (−0.45, −0.20)		−6.32 (−8.36, −4.27)	

Adjusted for age, sex, race, education level, marital status, alcohol intake, smoking status, leisure-time physical activity, BMI, the family of poverty ratio, TC, hypertension, diabetes, and CVD, if not stratified.

Supplementary Table 3. The relation of serum β-cryptoxanthin concentration with VAI or LAP in various subgroups.

β-cryptoxanthin	VAI		LAP	
	β (95% CI)	*P* for interaction	β (95% CI)	*P* for interaction
Age		0.725		0.307
<60	−0.29 (−0.47, −0.12)		−4.98 (−7.65, −2.30)	
⩾60	−0.24 (−0.35, −0.14)		−5.16 (−7.64, −2.68)	
Sex		0.880		0.328
Male	−0.28 (−0.38, −0.17)		−5.30 (−7.67, −2.93)	
Female	−0.26 (−0.48, −0.04)		−4.37 (−7.83, −0.92)	
BMI		0.722		0.087
<30	−0.28 (−0.45, −0.12)		−6.17 (−9.05, −3.30)	
⩾30	−0.31 (−0.50, −0.13)		−9.94 (−14.76, −5.12)	
Race		0.002		0.005
white	−0.41 (−0.59, −0.22)		−6.80 (−9.71, −3.88)	
non-white	−0.07 (−0.20, 0.07)		−1.42 (−4.01, 1.18)	
Current drinker		0.008		0.009
Now	−0.43 (−0.61, −0.24)		−7.87 (−10.37, −5.38)	
No	−0.10 (−0.23, 0.04)		−1.31 (−3.86, 1.24)	
Current smoker		0.115		0.238
Yes	−0.63 (−1.18, −0.09)		−8.88 (−16.95, −0.82)	
No	−0.20 (−0.30, −0.11)		−4.12 (−6.07, −2.18)	
Hypertension		0.915		0.351
Yes	−0.27 (−0.41, −0.13)		−5.91 (−8.94, −2.89)	
No	−0.27 (−0.46, −0.08)		−4.35 (−7.17, −1.53)	
CVD		0.497		0.619
Yes	−0.05 (−0.36, 0.27)		−1.46 (−7.95, 5.04)	
No	−0.30 (−0.45, −0.15)		−5.37 (−7.78, −2.96)	

Adjusted for age, sex, race, education level, marital status, alcohol intake, smoking status, leisure-time physical activity, BMI, the family of poverty ratio, TC, hypertension, diabetes, and CVD, if not stratified.

Supplementary Table 4. The relation of serum Lutein/zeaxanthin concentration with VAI or LAP in various subgroups.

Lutein/zeaxanthin	VAI		LAP	
	β (95% CI)	*P* for interaction	β (95% CI)	*P* for interaction
Age		0.062		0.006
<60	−0.26 (−0.43, −0.10)		−3.35 (−6.33, −0.37)	
⩾60	−0.33 (−0.43, −0.23)		−6.44 (−8.39, −4.48)	
Sex		<0.001		0.017
Male	−0.11 (−0.25, 0.02)		−1.42 (−4.45, 1.61)	
Female	−0.40 (−0.54, −0.26)		−7.03 (−9.47, −4.59)	
BMI		0.322		0.933
<30	−0.32 (−0.43, −0.22)		−6.61 (−8.46, −4.76)	
⩾30	−0.22 (−0.45, 0.01)		−7.90 (−13.76, −2.04)	
Race		0.004		0.008
white	−0.36 (−0.50, −0.23)		−5.86 (−8.38, −3.34)	
non-white	−0.06 (−0.21, 0.08)		−0.58 (−3.55, 2.39)	
Current drinker		0.621		0.965
Now	−0.30 (−0.44, −0.16)		−4.71 (−7.19, −2.22)	
No	−0.20 (−0.33, −0.07)		−3.09 (−5.76, −0.41)	
Current smoker		0.200		0.169
Yes	−0.23 (−0.75, 0.28)		−0.47 (−9.70, 8.76)	
No	−0.27 (−0.36, −0.17)		−4.76 (−6.76, −2.76)	
Hypertension		0.501		0.474
Yes	−0.22 (−0.33, −0.12)		−3.59 (−6.22, −0.96)	
No	−0.30 (−0.47, −0.12)		−4.59 (−7.58, −1.61)	
CVD		0.319		0.188
Yes	−0.25 (−0.47, −0.03)		−4.71 (−9.54, 0.11)	
No	−0.28 (−0.40, −0.16)		−4.32 (−6.48, −2.16)	

Adjusted for age, sex, race, education level, marital status, alcohol intake, smoking status, leisure-time physical activity, BMI, the family of poverty ratio, TC, hypertension, diabetes, and CVD, if not stratified.

Supplementary Table 5. The relation of serum Trans-lycopene concentration with VAI or LAP in various subgroups.

Trans-lycopene	VAI		LAP	
	β (95% CI)	*P* for interaction	β (95% CI)	*P* for interaction
Age		0.661		0.596
<60	−0.37 (−0.63, −0.11)		−6.74 (−10.16, −3.32)	
⩾60	−0.15 (−0.34, 0.03)		−2.78 (−6.85, 1.29)	
Sex		0.181		0.255
Male	−0.25 (−0.36, −0.14)		−5.65 (−8.29, −3.00)	
Female	−0.40 (−0.78, −0.03)		−6.52 (−11.23, −1.81)	
BMI		0.078		0.255
<30	−0.38 (−0.66, −0.11)		−6.10 (−9.54, −2.66)	
⩾30	−0.21 (−0.46, 0.04)		−7.20 (−12.21, −2.18)	
Race		0.097		0.081
white	−0.30 (−0.56, −0.04)		−5.15 (−8.48, −1.81)	
non-white	−0.43 (−0.62, −0.24)		−8.64 (−12.01, −5.27)	
Current drinker		0.851		0.639
Now	−0.34 (−0.61, −0.07)		−5.92 (−9.36, −2.47)	
No	−0.28 (−0.46, −0.10)		−5.72 (−9.49, −1.96)	
Current smoker		0.590		0.632
Yes	−0.52 (−1.17, 0.14)		−8.20 (−17.03, 0.63)	
No	−0.23 (−0.32, −0.13)		−4.66 (−6.70, −2.63)	
Hypertension		0.156		0.039
Yes	−0.17 (−0.35, 0.02)		−3.17 (−7.31, 0.97)	
No	−0.42 (−0.74, −0.10)		−7.41 (−11.27, −3.55)	
CVD		0.538		0.514
Yes	−0.09 (−0.57, 0.40)		−1.18 (−11.60, 9.24)	
No	−0.36 (−0.58, −0.14)		−6.50 (−9.27, −3.72)	

Adjusted for age, sex, race, education level, marital status, alcohol intake, smoking status, leisure-time physical activity, BMI, the family of poverty ratio, TC, hypertension, diabetes, and CVD, if not stratified.

Supplementary Table 6. The associations of the quartile of serum carotenoids, relative to Quartile 1 with VAI and LAP excluding current smokers (N=3919).

Categories	Range	VAI	LAP
		β (95%CI)	β (95%CI)
**α-carotene (ug/dL)**			
Continuous		−0.22 (−0.29, −0.15)	−4.45 (−6.01, −2.89)
Q1	<1.70	Ref.	Ref.
Q2	1.70–3.10	−0.16 (−0.43, 0.10)	−5.95 (−12.52, 0.62)
Q3	3.10–5.59	−0.48 (−0.76, −0.19)	−11.19 (−18.07, −4.31)
Q4	≥5.59	−0.68 (−0.94, −0.42)	−14.69 (−20.81, −8.57)
P for trend		<0.001	<0.001
**β-carotene (ug/dL)**			
Continuous		−0.28 (−0.37, −0.18)	−5.60 (−7.40, −3.79)
Q1	<8.70	Ref.	Ref.
Q2	8.70–14.70	−0.44 (−0.71, −0.18)	−11.52 (−18.03, −5.02)
Q3	14.70–25.88	−0.67 (−0.96, −0.37)	−16.17 (−23.08, −9.27)
Q4	>25.88	−1.12 (−1.40, −0.84)	−24.45 (−31.06, −17.84)
P for trend		<0.001	<0.001
**β-cryptoxanthin (ug/dL)**			
Continuous		−0.21 (−0.31, −0.11)	−4.30 (−6.33, −2.27)
Q1	<5.40	Ref.	Ref.
Q2	5.40–8.60	−0.18 (−0.43, 0.06)	−3.93 (−9.61, 1.75)
Q3	8.60–13.84	−0.44 (−0.70, −0.19)	−9.27 (−15.17, −3.37)
Q4	>13.84	−0.56 (−0.80, −0.31)	−10.67 (−16.19, −5.16)
P for trend		<0.001	<0.001
**Lutein/zeaxanthin (ug/dL)**			
Continuous		−0.28 (−0.38, −0.18)	−4.94 (−7.01, −2.86)
Q1	<11.20	Ref.	Ref.
Q2	11.20–15.50	−0.32 (−0.53, −0.11)	−4.39 (−9.69, 0.90)
Q3	15.50–21.10	−0.52 (−0.77, −0.27)	−9.82 (−15.31, −4.33)
Q4	≥21.10	−0.68 (−0.98, −0.38)	−11.60 (−18.08, −5.12)
P for trend		<0.001	<0.001
**Trans-lycopene (ug/dL)**			
Continuous		−0.23 (−0.33, −0.13)	−4.72 (−6.78, −2.66)
Q1	<14.10	Ref.	Ref.
Q2	14.10–20.20	−0.26 (−0.51, −0.01)	−5.82 (−10.38, −1.26)
Q3	20.20–28.60	−0.35 (−0.59, −0.11)	−6.55 (−11.94, −1.16)
Q4	≥28.60	−0.66 (−0.98, −0.34)	−13.61 (−20.17, −7.05)
P for trend		<0.001	<0.001

Adjusted for age, sex, race, education level, marital status, alcohol intake, leisure-time physical activity, BMI, the family of poverty ratio, TC, hypertension, diabetes, and CVD.
